# Neuroimaging oxytocin modulation of social reward learning in schizophrenia

**DOI:** 10.1192/bjo.2022.577

**Published:** 2022-09-26

**Authors:** Elias D. Mouchlianitis, Derek K. Tracy, Rebekah Wigton, Lucy D. Vanes, Anne-Kathrin Fett, Sukhi S. Shergill

**Affiliations:** Cognition, Schizophrenia and Imaging Laboratory, Institute of Psychiatry, Psychology & Neuroscience, King's College London, UK; and School of Psychology, University of East London, UK; Cognition, Schizophrenia and Imaging Laboratory, Institute of Psychiatry, Psychology & Neuroscience, King's College London, UK; and West London NHS Trust, London, UK; Department of Neurology, Beth Israel Deaconess Medical Center, Boston, Massachusetts, USA; Cognition, Schizophrenia and Imaging Laboratory, Institute of Psychiatry, Psychology & Neuroscience, King's College London, UK; Department of Psychology, City University of London, UK; Cognition, Schizophrenia and Imaging Laboratory, Institute of Psychiatry, Psychology & Neuroscience, King's College London, UK; Kent and Medway Medical School, University of Kent, Canterbury, UK; and Kent and Medway NHS and Social Care Partnership Trust, Gillingham, UK

**Keywords:** Oxytocin, psychosis, schizophrenia, fMRI, trust

## Abstract

**Background:**

Conventional pharmacological approaches have limited effectiveness for schizophrenia. There is interest in the application of oxytocin, which is involved in social cognition. Clinical trials have yielded mixed results, with a gap in understanding neural mechanisms.

**Aims:**

To evaluate the behavioural impact of oxytocin administration on a social learning task in individuals with schizophrenia, and elucidate any differential neural activity produced.

**Method:**

We recruited 20 clinically stable right-handed men diagnosed with schizophrenia or schizoaffective disorder. In a double-blind cross-over randomised controlled study, 40 IU of oxytocin or placebo were administered before functional magnetic resonance imaging of participants playing a multi-round economic exchange game of trust. Participants had the role of investors (investment trials) receiving repayment on their investments (repayment trials), playing one session against a computer and a second against a player believed to be human.

**Results:**

During investment trials, oxytocin increased neural signalling in the right lateral parietal cortex for both human and computer player trials, and attenuated signalling in the right insula for human player trials. For repayment trials, oxytocin elicited signal increases in left insula and left ventral caudate, and a signal decrease in right amygdala during the human player trials; conversely it resulted in right dorsal caudate activation during the computer player trials. We did not find a significant change in behavioural performance associated with oxytocin administration, or any associations with symptoms.

**Conclusions:**

During a social learning task oxytocin modulates cortical and limbic substrates of the reward-processing network. These perturbations can be putatively linked to the pathoaetiology of schizophrenia.

Schizophrenia is characterised by a complex interplay of positive and negative symptoms, and cognitive dysfunction. All have a marked impact on social and occupational functioning, with such deficits typically appearing before a diagnosis is first made.^1^ Although antipsychotic medication can attenuate positive symptoms, it is ineffective on other symptom domains, which typically have greater impact on individuals’ quality of life.^2^

Oxytocin is a neuropeptide that regulates social bonding and improves social cognition across numerous domains.^3^ In healthy participants, its administration has been shown to modulate activity in an extended network of regions involved in: mentalising, such as the medial prefrontal cortex, temporoparietal junction, middle and superior temporal cortex; reward processing, such as the dorsal and ventral striatum; and emotion processing, such as the amygdala and insula.^4^ Oxytocin has been studied as a potential prosocial intervention in schizophrenia. To date, clinical studies have yielded mixed results, as two meta-analyses did not report any difference between placebo and oxytocin in the attenuation of positive and negative symptoms.^5^ Note, however, that Oya et al^5^ reported improvements on the General dimension of the Positive and Negative Syndrome Scale (but not Total PANSS), which suggests that oxytocin can affect certain dimensions of symptomatology. These mixed findings highlight the challenges for meta-analytical methods in comparing trials with varying clinical populations, and the breadth of ways ‘social cognition’ can be understood and evaluated.^6^ Green et al^7^ note both the growing literature on non-social and social cognition and the neuroimaging of underlying substrates, and the complexity of issues that belie more simplistic explanations of functioning: from illness heterogeneity, through impairments varying across the lifespan, to the variety of pharmacological, psychological and cognitive remediating interventions trialled to measure and/or improve functioning. Added to this, other neuropeptides and hormonal systems are clearly of relevance, potentially in complex interacting ways. Of particular note in this regard, the vasopressin system has been intimately linked with both oxytocin and sociality and emotional behaviours. Further, meta-analytical works still emphasise an underpinning lack of understanding of the mechanisms through which oxytocin influences behaviour, cognition and any other symptomatology. Nevertheless, better specificity is required, including linking behavioural and/or clinical changes with any underlying neural alterations.

As noted, one of these putative targets is aberrant reward processing. This has appeal as an area of study as it is one of the core processing deficits in schizophrenia, and one with elements that bridge symptom domains.^8^ It lends itself to a combined behavioural–neuroimaging approach with relative specificity, although of course administration of oxytocin may also affect related and potentially confounding processes such as emotion processing, mentalising and attribution. One method of evaluating reward value and motivational salience is via trust and reciprocity, which can be measured in an economic exchange.^9^ These studies demonstrate that trust can be operationalised as a function of motivational salience, where the trusting behaviours are dependent on the inference of perceived incentive or aversive salience outcomes. Importantly, these are processes that have been shown to be previously modulated by oxytocin.^4^

Thus, the trust game is employed in the present study to investigate these processes in schizophrenia. Studies using this task have found that people with schizophrenia showed decreased trust behaviourally^10^ and a markedly reduced response in reward-processing regions such as the caudate, temporoparietal junction and parietal cortex.^11^ Gromann et al^11^ reported that caudate signal reductions were associated with increased persecutory delusions, suggesting a link between neural correlates of trust and symptomatology.

Our aim was to employ functional magnetic resonance imaging (fMRI) to measure the neural effects of the administration of oxytocin on social reward learning in individuals with schizophrenia. In terms of brain activation, we focused on the key subcortical reward-processing regions (striatum and amygdala) and cortical regions (orbitofrontal cortex, prefrontal cortex, parietal cortex, insula) activated when explicit reward signals need to be integrated with sensory and contextual signals.^12^ We expected that oxytocin would elicit signal increases in these regions, and hypothesised that oxytocin would increase behavioural measures of trust.

## Method

### Participants

Twenty right-handed males with schizophrenia or schizoaffective disorder, diagnosed according to the ICD-10, participated in this study. IQ was estimated using the two-item Wechsler Abbreviated Scale of Intelligence (WASI), consisting of the vocabulary and matrix reasoning subtests. Nineteen of the participants were taking antipsychotic medication throughout the course of the study (olanzapine: *n* = 11; risperidone: *n* = 3; fluphenazine decanoate: *n* = 1; zuclopenthixol decanoate: *n* = 1; clozapine: *n* = 3; haloperidol: *n* = 1). All participants signed informed consent forms and were compensated for their participation in the study on completion of the testing. The authors assert that all procedures contributing to this work comply with the ethical standards of the relevant national and institutional committees on human experimentation and with the Helsinki Declaration of 1975, as revised in 2008. Ethical approval was obtained from the Camberwell and St Giles Research Ethics Committee (ethics approval number 87370).

### Oxytocin administration protocol

The study used a randomised double-blind cross-over design in administering oxytocin and placebo. Both the oxytocin (40 IU) and matched placebo nasal sprays were self-administered by our participants. Each participant was told to tilt their head back at a slight angle and insert the nasal spray into one of their nostrils while trying to keep the spray bottle as upright as possible. They were then told to push down on the pump mechanism while simultaneously inhaling through their nostrils as deeply as possible. They were then asked to switch nostrils and repeat this administration. Following a protocol previously employed, a break of 45 s was given between each administration to allow for the nasal spray to be absorbed by the nostrils.^13^ Proper administration was demonstrated to each participant before self-administration. Each spray was dispensed on the day of administration by the South London and Maudsley pharmacy, approximately 1 h before being administered, ensuring optimal storage temperature prior to administration. Oxytocin/placebo administration was arranged to take place 45 min before the start of the first task within the fMRI scanner. This was in line with previous fMRI studies which have shown significant changes in neural activity after oxytocin administration using this same time frame.^14^ Participants were scanned for each condition a week apart.

### fMRI acquisition

The fMRI data were acquired on a Discovery MR750 3 T scanner at the Centre for Neuroimaging Sciences, King's College London (T_2_*-weighted gradient-echo echo-planar images (EPIs), repetition time TR = 2000 ms, echo time TE = 35 ms, flip angle 75°, 64 × 64 matrix, 24 cm field of view). A 12-phase head coil array was used over the whole head for radiofrequency (RF) transmission and reception. Each whole-brain image contained 38 3 mm axial slices separated by a distance of 0.3 mm with in-plane isotropic voxel resolution of 3.75 × 3.75 mm. Two sessions were recorded for each participant (374 volumes for each session).

Before the experimental portion of each session, a T_1_-weighted structural scan using a fast-spoiled gradient-echo pulse sequence (TR = 9.356 ms, TE = 3.828 ms, flip angle 12°, time to inversion 450 ms) was acquired for reference purposes. The first four volumes were discarded to allow for transient effects. Participants made their responses using two buttons on a button box with the index and middle fingers of their right hand. Head movement was minimised using headphones and additional padding around the head and ears as well as around the arms and legs.

### fMRI task

The trust game consisted of a modified version of a previous multi-round trust game.^9^ There were two different sessions: one where they were explicitly informed that they would be playing against a computer and another where they were led to believe they were playing against another human player. In fact, both opponents were a computer program using the same algorithm of a cooperative investment style.

The ‘human’ players were represented by randomised initials, to give the impression of playing an opponent without bias towards gender or specific names. Participants played the role of the first player. Each session of the task (human player and computer player) consisted of 20 real trials and 20 control trials. The design and duration of each event within the control trials was identical to the game trials. However, in each of the control trials, participants were told to move the cursor denoting donation amount to a specific number which was highlighted with a red arrow. Participants were told that the control trials were not related to investment decisions. All participants performed a number of practice rounds to ensure that they understood the task.

At the beginning of each round, participants received the same starting budget of £10. Any amount between £0 and £10 could be shared. Then the first repayment was either 100%, 150% or 200% of the invested amount, each with a probability of 33%. For this study, we employed a cooperative player style. Repayments increased probabilistically if there was an increase relative to the previous investment but remained stable in all other situations. For each increase in investment, the chance of a maximum repayment of 200% increased by 10% and the chance of a minimum repayment of 100% decreased by 10%. Every trial started with an investment cue of £10 and a maximum of 6 s, during which the participant had to make their investment. The invested amount was shown (2 s), followed by a waiting period with a bar slowly filling itself with dots (2–4 s), and a fixation cross (500 ms). During this time, the cursor started at £5 and participants had to select any other amount by pressing up or down with the button box. If no response was made, the investment defaulted to £5. The partner's response was displayed (3 s), followed by the totals (2–4 s depending on the length of the partner's response). Each trial ended with a fixation cross (500 ms). In total, each trial lasted 18.5 s.

### Statistical analyses

#### Behavioural analysis

The two main behavioural experimental measures were: (a) the initial investment (i.e. baseline trust in the other player); (b) mean investment across the 20 experimental trials. Each measure was analysed using repeated-measures two-way analysis of variance (two-tailed, *P* < 0.05), with the factors Drug (Placebo versus Oxytocin) and Player (Human versus Computer).

#### fMRI analysis

##### First-level analysis

The fMRI data analysis was carried out using a general linear model as implemented in FEAT (FMRI Expert Analysis Tool) Version 6.00, part of FSL (FMRIB's Software Library, www.fmrib.ox.ac.uk/fsl), on Microsoft Windows. Functional and structural brain images were extracted from non-brain tissue using FSL's brain extraction tool (BET), and EPI images were realigned using MCFLIRT to correct effects of head motion. A 100 s temporal high-pass filter was applied and data were spatially smoothed using a Gaussian kernel of 6 mm full width at half maximum (FWHM). For the first-level analysis, the investment and repayment phases of the experimental and control trials of the task were modelled separately, as reward learning and trust perception might shift from outcome to anticipation while a model of the partner reciprocal is built over trials. Contrasts of interest for each participant were created by comparing mean blood oxygen level-dependent (BOLD) signal of investment and repayment trials with their respective control trials. The design matrix also included six standard motion parameters as well as a motion artifact confound matrix, which identified and regressed motion-corrupted volumes. Volumes detected as corrupted were calculated using the DVARS metric as implemented by FSL Motion Outliers in FSL (https://fsl.fmrib.ox.ac.uk/fsl/fslwiki/FSLMotionOutliers). Participants were excluded if the number of motion-corrupted volumes for either the placebo or the oxytocin scanning session was above two standard deviations from the group mean. No participants were excluded from the trial based on either of these criteria.

##### Second-level analysis

For the second-level analysis we focused on key brain regions responsible for reward learning and motivational salience, defined in a seminal review on reward processing by Schultz.^12^ We created a binary mask from regions of interest (ROIs) defined *a priori* that included the dorsolateral prefrontal cortex, orbitofrontal cortex, parietal cortex, striatum and amygdala. The insula was also included as substantial evidence shows significant effects of oxytocin in that region.^3^ The striatal mask was created by combining the caudate and putamen. The ROIs were defined bilaterally from the probabilistic structural Harvard–Oxford MNI (Montreal Neurological Institute) atlases in FSL, thresholded at 50% probability. Initially, we investigated effects in these regions using a two-way factorial design with the factors Drug (Placebo versus Oxytocin) and Player (Human versus Computer). Significance was defined using family-wise error (FWE) small-volume correction for regions within the structural mask at *P* < 0.05, at a cluster-forming threshold of *Z* > 2.3. To balance between sensitivity and validity, we also ran separate analyses for human and computer player sessions, using small-volume correction for each ROI with a cluster-forming threshold of Z > 2.7 at *P* < 0.001 and cluster extent larger than 40 voxels.

## Results

The demographic and clinical characteristics of the participants are summarised in Table 1.

**Table 1:** Demographic and clinical characteristics of participants (*n* = 20)

Characteristic	
Age, years: mean (s.d.)	37.90 (7.43)
WASI score, mean (s.d.)	98.85 (13.24)
NS-SeC, mean (s.d.)	2.80 (1.70)
Gender, male: *n*	20
Age at onset, years: mean (s.d.)	24.50 (6.48)
Duration of illness, years: mean (s.d.)	13.40 (6.53)
CPZ equivalents, mean (s.d.)	430.05 (236.27)
PANSS Positive symptom score, mean (s.d.)	14.95 (4.97)
PANSS Negative symptom score, mean (s.d.)	18.30 (4.99)
PANSS General symptom score, mean (s.d.)	30.70 (7.10)
PANSS Total score, mean (s.d.)	63.95 (14.56)

WASI, Wechsler Abbreviated Scale of Intelligence; NS-SeC, National Statistics Socio-economic Classification; CPZ, chlorpromazine; PANSS, Positive and Negative Syndrome Scale.

### Behavioural results

We did not find a significant interaction or significant main effects for initial investments. For mean investments, the Drug × Player interaction approached significance: *F*(1,19) = 4.03, *P* = 0.06. This interaction was driven by a higher mean investment during oxytocin administration for human player trials compared with computer trials; after placebo administration the difference between human and computer player trials was not significant (Table 2).

**Table 2:** Behavioural results for first investment and mean investment per condition

Measure and player	Drug	
	Placebo	Oxytocin
First investment, £: mean (s.d.)		
Human	5.50 (3.2)	5.40 (2.9)
Computer	6.70 (2.8)	5.85 (2.8)
Mean investment, £: mean (s.d.)		
Human	6.04 (2.04)	6.12 (2.57)
Computer	6.16 (2.15)	5.13 (2.42)

### Structural mask analysis

#### Investment trials

**Fig. 1:**
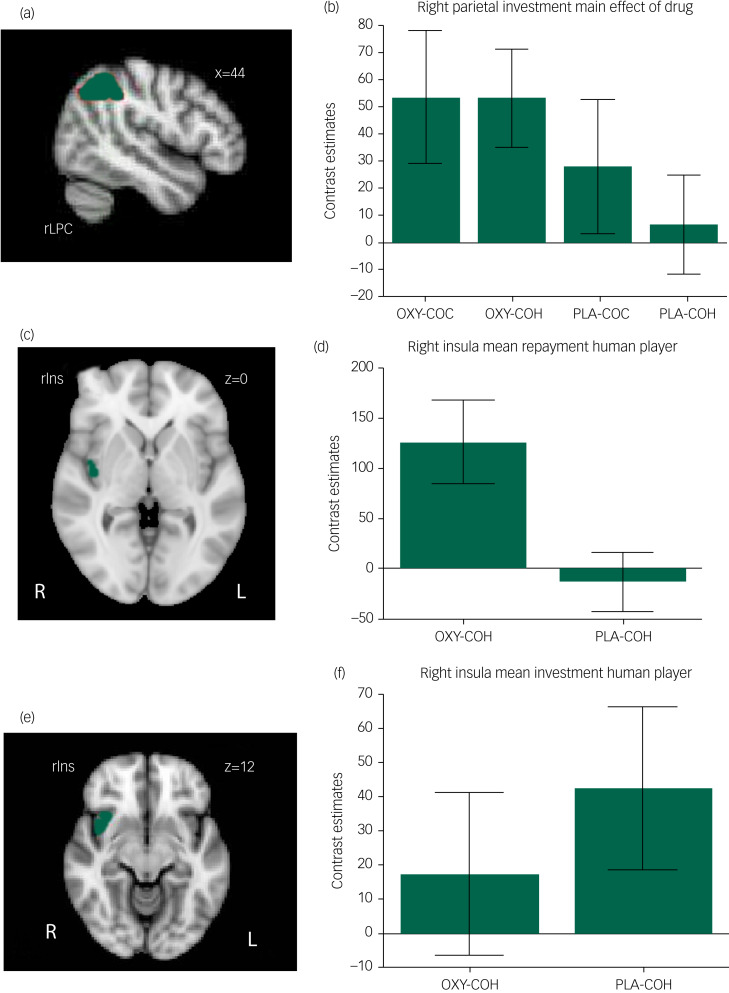
Oxytocin effects in the right parietal lobule and insular cortex. (a) The cluster in the inferior right parietal lobule that was significantly modulated by oxytocin compared with placebo during investment trials (family-wise-error corrected *P* < 0.05). (c) and (e) Clusters that were modulated significantly by oxytocin compared with placebo in the insula for repayment trials (c) and investment trials (e) (cluster-forming threshold *Z* > 2.7, cluster extend threshold *k* > 40, significance threshold *P* < 0.001, uncorrected). (b), (d) and (f) Mean activation from corresponding regions for the paired *t*-test (two-tailed), *P* < 0.05. Error bars show standard error of the mean. OXY, oxytocin; PLA, placebo; COC, cooperative computer player; COH, cooperative human player.

The Drug × Player factorial analysis revealed a significant main effect of Drug in a right lateral parietal cluster (MNI: *x* = 44, *y* = −62, *z* = 44, *k* = 48, *Z =* 4.63, *P* < 0.001, FWE corrected). Oxytocin administration increased activation during investment trials for both the human and computer sessions (Fig. 1a and b). No region demonstrated a significant interaction or main effect of Player.

#### Repayment trials

No significant interaction or main effects were found for repayment trials.

Hereafter, all ROI analyses (excluding the parietal cortex ROI above with FWE correction) are reported at a cluster-forming threshold of *Z* > 2.7 at a significance level of *P* < 0.001 and cluster extent larger than 40 voxels.

### Insula ROI analysis

#### Investment trials

Oxytocin significantly attenuated neural activity during investment trials for the human session in a right anterior insula cluster (Z = 2.62, *P* < 0.001, Fig. 1c and d), but no significant differences were found for the computer session.

#### Repayment trials

A cluster in the right insula showed increased activation after oxytocin administration for the human session (MNI: *x* = 42, *y* = −8, *z* = 0, *k* = 42, *Z* = 4.33, *P* < 0.001, Fig. 1e and f), but no significant differences were found for the computer session.

### Striatal ROI analysis

#### Investment trials

No differences were found for the investment trials for either Drug or Player conditions.

#### Repayment trials

**Fig. 2:**
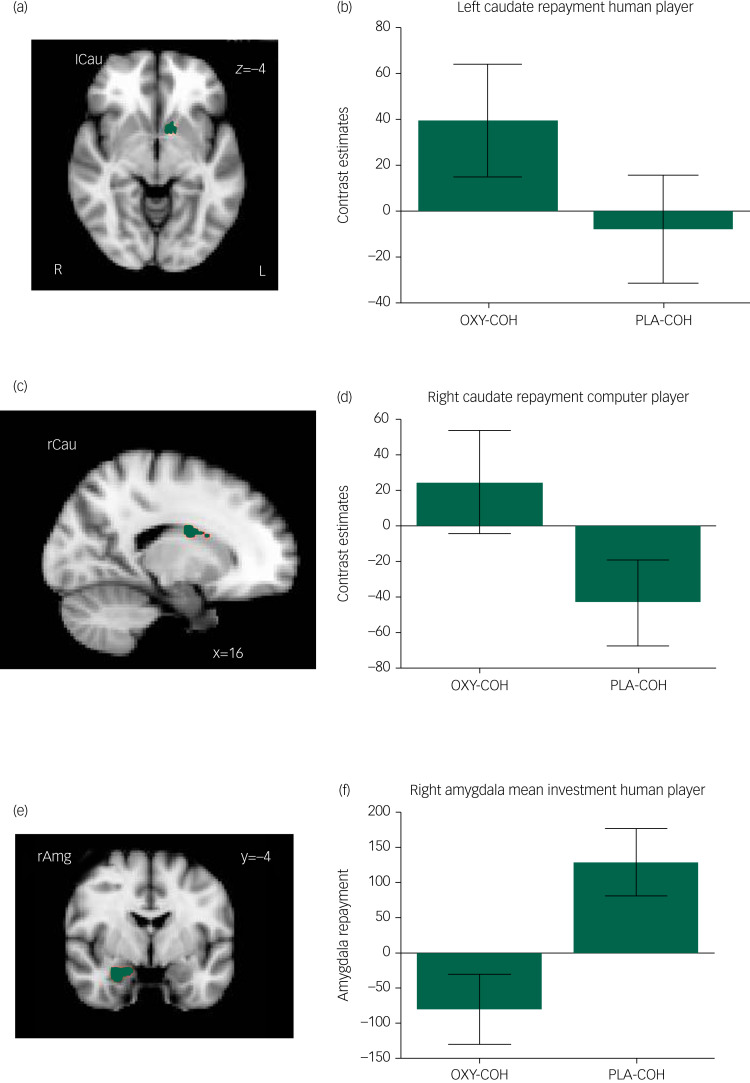
Oxytocin effects in the caudate and amygdala. (a), (c) and (e) Clusters that were modulated significantly by oxytocin compared with placebo in the striatum and amygdala during repayment trials (cluster-forming threshold *Z* > 2.7, cluster extend threshold *k* > 40, significance threshold *P* < 0.001). (b), (d) and (f) Mean activation from corresponding regions for the paired *t*-test (two-tailed), *P* < 0.05. Error bars show standard error of the mean. OXY, oxytocin; PLA, placebo; COC, cooperative computer player; COH, cooperative human player.

For the repayment trials oxytocin significantly increase activation relative to the placebo in a cluster in the left ventral caudate when playing against the human player (MNI: *x* = −10, *y* = −6, *z* = −6, *k* = 43, Z = 2.7, *P* < 0.001, Fig. 2a and b) and right dorsal caudate cluster when playing against the computer player (MNI: *x* = 16, *y* = −4, *z* = 42, *k* = 48, *Z* = 2.7, *P* < 0.001, Fig. 2c and d).

### Amygdala ROI analysis

#### Investment trials

No significant effects were found during the investment trials for either the human or computer sessions.

#### Repayment trials

Oxytocin significantly attenuated amygdala activity during the human sessions within a cluster in the right amygdala (MNI: *x* = 28, *y* = −4, *z* = −26, *k* = 74, Z = 3.09, *P* < 0.001, Fig. 2e and f).

### Associations with symptoms

None of the ROIs or behavioural measures showed any association with symptoms (*P* > 0.05).

## Discussion

Oxytocin administration to individuals with schizophrenia induced signal changes in a number of brain regions related to trust. The pattern of activations observed is consistent with the reward-processing literature regarding explicit and implicit reward signalling.^12^ Explicit reward signals, operationalised in the trust task as repayments, are processed in limbic substrates of the reward-processing network such as the striatum and the amygdala. Implicit and contextual reward signals, operationalised as investments, are processed by cortical substrates of the reward-processing network. Our findings are consistent with this account. Oxytocin does not seem to have a global effect in modulating reward-processing substrates, but it modulates cortical or subcortical substrates depending on whether investment or repayment trials were processed.

More specifically, the most robust effect of oxytocin was observed as increased signalling in the inferior lateral parietal lobe, a key cortical substrate of attention, monitoring and updating of reward processing, particularly within the context of motivational salience.^15^ A critical role of the lateral parietal cortex is forming action–outcome associations in reward learning, by encoding the history of previous choices and their reward value, as well as calculating potential outcomes of alternative actions to the ones taken.^16^ These processes are relevant to the trust task used in the present experiment. More specifically, the participant is required to create a model of reciprocal behaviour based on the history of repayments received that would allow forming decisions about the investment style of the other player. Thus, dysfunction in the parietal cortex can result in aberrant encoding and processing of this information, which can lead to suboptimal investment choices.

Oxytocin also significantly modulated insular cortex activation during the human player session. We found signal reductions during investment and signal increases during repayment. This is consistent with the established role of the insula in social and emotional cognition but also in trusting behaviour specifically.^17^ The insula is also engaged under conditions of motivational salience,^18^ which is a key process required to infer the repayment style of the other player in the trust game (in the case of our experiment, a cooperative style).

In the caudate, oxytocin administration increased signalling during both the human and computer sessions in the repayment trials. The caudate is a key substrate of the reward-processing system, coding both the anticipated and received reward^19–21^ and the contingencies between actions and outcomes.^22^ Other work involving the trust game have shown caudate activation to increase with more generous repayments in healthy participants.^9^ Individuals with schizophrenia have previously shown significant signal reductions during cooperative repayments compared with healthy controls.^23,11^ This likely reflects aberrant social reward processing and failure to encode positive outcomes correctly, distorting perceptions of reciprocity and decreasing trust. Gromann et al^11^ also found that reduced caudate responses to repayment were associated with increased persecutory ideations. Hence, in this current work, the upregulation of caudate signal in response to oxytocin during the repayment trials suggests normalisation of signalling in relation to expected and perceived reward.

Oxytocin elicited a reduction of neural activation in the amygdala during repayment trials relative to placebo for the human player condition. This is consistent with previous findings that oxytocin reduced amygdala reactivity in similar trust tasks in healthy participants^4,24^, which in turn is in keeping with reported amygdalar responses to reward signals with affective significance and encoding motivational salience.^25^

The effects of oxytocin in these core cortical and subcortical reward-processing regions suggest a plausible neurobiological pathway for potentially ameliorating social deficits and paranoia in schizophrenia. All current atypical antipsychotic drugs owe their efficacy to the regulation of the dopamine neurons predominantly in the striatum, together with the modulation of serotonin interneurons cortically and subcortically. The amygdala and its corticolimbic connections are densely innervated presynaptically by dorsal raphe nucleus 5-HT neurons.^27^ Recent evidence has shown that oxytocin modulates dopamine binding in the dorsal striatum^26,27^ and serotonin binding in the amygdala.^28,29^ Furthermore, the medial amygdala network that mediates social behaviour and bonding is modulated by midbrain and striatal dopamine, and the strength of the modulation also shows a significant association with oxytocin levels.^30^ Our data suggest that, even at a single dose, intranasal oxytocin modulates disrupted signalling within an extended neural network of cortical and subcortical substrates involved in reward processing and social cognition.

### Implications and limitations

Our results are interesting in the context of wider therapeutic applications of oxytocin in schizophrenia. Given it has a modulatory effect on limbic and cortical structures that are dysfunctional and are targeted by current antipsychotic medication, it might act synergistically to regulate perturbations in these regions to further improve the efficacy of antipsychotics. For example, one potential application could be as an adjunctive intervention for patients who show poor antipsychotic response and who typically appear to have normal dopaminergic function.^31,32^ Clozapine, the only effective antipsychotic for treatment-resistant schizophrenia, has low affinity for striatal dopamine receptors, but high affinity for cortical D_1_ receptors, as well as *N*-methyl-d-aspartate (NMDA), cholinergic and gamma-aminobutyric acid (GABA) receptors in the cortex.^33^ It is noteworthy that regions we find in our study to be modulated by oxytocin are also commonly regulated by clozapine administration.^34^ Oxytocin has not been tested in patients with treatment-resistant schizophrenia yet, but these data suggest possibly increased efficacy in such individuals.

Our study has several limitations. First, we did not directly measure oxytocin plasma concentrations, although previous research has shown that the protocol we used adequately raises plasma levels to modulate cognitive function and BOLD signal. Second, we did not measure the effects of oxytocin in a matched healthy control group, which would allow us to draw disease-specific conclusions. Third, our study had a relatively small sample size, only included male participants, and some work has shown differential effects of oxytocin in healthy male and female participants during reward processing.^35^ Added to this is the potential influence of other neuropeptide and hormonal signalling. In a male-only sample, testosterone putatively might play a role: although not measured in this group, there are data linking raised levels with reduced social cognition. Further, as noted in the introductory paragraphs, vasopressin is intimately linked with oxytocin and social functioning. Future work might usefully provide measurements of these potential confounders. The sample size for the study was derived from the study by Gromann et al,^11^ which investigated the neural correlates of trust in schizophrenia. Although the sample size of the present study was adequate to reveal oxytocin-related modulation of cortical and subcortical regions during a trust task in people with schizophrenia, the study might have been underpowered to detect significant behavioural effects. The issue of gender is particularly pertinent, given the rich literature on differences by gender in social learning and reward. Future work should explore both the effects in females and evaluate any gender differences. Fourth, we did not find any association with symptoms. The patients we tested were stable and medicated. It is possible that the relatively narrow range of symptom scores within this group reduced the power to detect correlations. Further, the patients were on differing antipsychotics and dose regimens; the limited sample size was unable to control for these variables. Future work should include patients with a wider range of symptoms (e.g. including those resistant to antipsychotics) to increase statistical power, have adequate numbers to control for varying medication type and dose, have a matched female sample, and include measurements of other neuropeptides and hormones associated with emotional and social behaviour. Fifth, behavioural changes as a result of oxytocin administration only produced a trend-level interaction for mean investments, driven by a reduction of investment in the oxytocin computer player condition. There is evidence that oxytocin potentially increases in-group trust but decreases out-group trust as well.^36^ Putatively, knowledge of playing against a computer might have created such a bias. A single dose of oxytocin has acute and transient effects that upregulate key reward-processing substrates. Given that individuals with schizophrenia typically have reward-processing deficits, a single dose might not normalise these deficits sufficiently to elicit an impactful behavioural change. This might be further exacerbated by the fact that the participants in our study were clinically stable. Finally, although we targeted reward processing, oxytocin may have affected other aspects of social cognition, such as emotion processing, mentalising and attribution, all of which might have confounded the findings, and in particular the behavioural results.

The oxytocin-modulated perturbations in cortical and subcortical substrates revealed in our study can be argued to be associated with the aetiology of schizophrenia, although this remains somewhat speculative at this time, and the role of oxytocin in such pathophysiology remains an area of debate. However, as our work did not show any significant behavioural or symptom change from oxytocin administration, further research is needed to elucidate whether there are parameters whereby externally administered oxytocin might produce such changes and might be clinically beneficial.^37^

## Data Availability

The data that support the findings of this study are available on request from the corresponding author E.D.M. The data are not publicly available as they contain information that could compromise the privacy of research participants.

## References

[1] Fett AKJ, Shergill SS, Krabbendam L. Social neuroscience in psychiatry: unravelling the neural mechanisms of social dysfunction. Psychol Med 2015; 45: 1145–65. 25335852 10.1017/S0033291714002487

[2] Penn DL, Keefe RSE, Davis SM, Meyer PS, Perkins DO, Losardo D, The effects of antipsychotic medications on emotion perception in patients with chronic schizophrenia in the CATIE trial. Schizophr Res 2009; 115: 17–23. 19766459 10.1016/j.schres.2009.08.016 PMC2765056

[3] Wigton R, Radua J, Allen P, Averbeck B, Meyer-Lindenberg A, McGuire P, Neurophysiological effects of acute oxytocin administration: systematic review and meta-analysis of placebo-controlled imaging studies. J Psychiatry Neurosci 2015; 40: e1–22. 25520163 10.1503/jpn.130289 PMC4275335

[4] Baumgartner T, Heinrichs M, Vonlanthen A, Fischbacher U, Fehr E. Oxytocin shapes the neural circuitry of trust and trust adaptation in humans. Neuron 2008; 58: 639–50. 18498743 10.1016/j.neuron.2008.04.009

[5] Oya K, Matsuda Y, Matsunaga S, Kishi T, Iwata N. Efficacy and safety of oxytocin augmentation therapy for schizophrenia: an updated systematic review and meta-analysis of randomized, placebo-controlled trials. Eur Arch Psychiatry Clin Neurosci 2016; 266: 439–50. 26303414 10.1007/s00406-015-0634-9

[6] Goh KK, Chen CH, Lane HY. Oxytocin in schizophrenia: pathophysiology and implications for future treatment. Int J Mol Sci 2021; 22(4): 2146. 33670047 10.3390/ijms22042146 PMC7926349

[7] Green MF, Horan WP, Lee J. Nonsocial and social cognition in schizophrenia: current evidence and future directions. World Psychiatry 2019; 18: 146–61. 31059632 10.1002/wps.20624 PMC6502429

[8] Hanssen E, van der Velde J, Gromann PM, Shergill SS, de Haan L, Bruggeman R, Neural correlates of reward processing in healthy siblings of patients with schizophrenia. Front Hum Neurosci 2015; 9: 504. 26441601 10.3389/fnhum.2015.00504 PMC4585217

[9] King-Casas B, Tomlin D, Anen C, Camerer CF, Quartz SR, Montague PR. Getting to know you: reputation and trust in a two-person economic exchange. Science 2005; 308: 78–83. 15802598 10.1126/science.1108062

[10] Fett AKJ, Shergill SS, Joyce DW, Riedl A, Strobel M, Gromann PM, To trust or not to trust: the dynamics of social interaction in psychosis. Brain 2012; 135(3): 976–84. 22366802 10.1093/brain/awr359

[11] Gromann PM, Heslenfeld DJ, Fett AK, Joyce DW, Shergill SS, Krabbendam L. Trust versus paranoia: abnormal response to social reward in psychotic illness. Brain 2013; 136: 1968–75. 23611807 10.1093/brain/awt076

[12] Schultz W. Neuronal reward and decision signals: from theories to data. Physiol Rev 2015; 95: 853–951. 26109341 10.1152/physrev.00023.2014 PMC4491543

[13] Guastella AJ, Hickie IB, McGuinness MM, Otis M, Woods EA, Disinger HM, Recommendations for the standardisation of oxytocin nasal administration and guidelines for its reporting in human research. Psychoneuroendocrinology 2013; 38: 612–25. 23265311 10.1016/j.psyneuen.2012.11.019

[14] Domes G, Heinrichs M, Gläscher J, Büchel C, Braus DF, Herpertz SC. Oxytocin attenuates amygdala responses to emotional faces regardless of valence. Biol Psychiatry 2007; 62: 1187–90. 17617382 10.1016/j.biopsych.2007.03.025

[15] Peck CJ, Jangraw DC, Suzuki M, Efem R, Gottlieb J. Reward modulates attention independently of action value in posterior parietal cortex. J Neurosci 2009; 29 11182–91. 19741125 10.1523/JNEUROSCI.1929-09.2009 PMC2778240

[16] Seo H, Barraclough DJ, Lee D. Lateral intraparietal cortex and reinforcement learning during a mixed-strategy game. J Neurosci 2009; 29: 7278–89. 19494150 10.1523/JNEUROSCI.1479-09.2009 PMC2743508

[17] Belfi AM, Koscik TR, Tranel D. Damage to the insula is associated with abnormal interpersonal trust. Neuropsychologia 2015; 71: 165–72. 25846668 10.1016/j.neuropsychologia.2015.04.003 PMC4417431

[18] Uddin LQ. Salience processing and insular cortical function and dysfunction. Nat Rev Neurosci 2014; 16: 55–61. 25406711 10.1038/nrn3857

[19] Hollerman JR, Tremblay L, Schultz W. Influence of reward expectation on behavior-related neuronal activity in primate striatum. J Neurophysiol 1998; 80: 947–63. 9705481 10.1152/jn.1998.80.2.947

[20] Hassani OK, Cromwell HC, Schultz W. Influence of expectation of different rewards on behavior-related neuronal activity in the striatum. J Neurophysiol 2001; 85(6): 2477–89. 11387394 10.1152/jn.2001.85.6.2477

[21] Cromwell HC, Schultz W. Effects of expectations for different reward magnitudes on neuronal activity in primate striatum. J Neurophysiol 2003; 89: 2823–38. 12611937 10.1152/jn.01014.2002

[22] Kimchi EY, Laubach M. The dorsomedial striatum reflects response bias during learning. J Neurosci 2009; 29: 14891–902. 19940185 10.1523/JNEUROSCI.4060-09.2009 PMC6666004

[23] Gromann PM, Shergill SS, de Haan L, Meewis DGJ, Fett AKJ, Korver-Nieberg N, Reduced brain reward response during cooperation in first-degree relatives of patients with psychosis: an fMRI study. Psychol Med 2014; 44: 3445–54. 25065732 10.1017/S0033291714000737

[24] Rilling JK, DeMarco AC, Hackett PD, Thompson R, Ditzen B, Patel R, Effects of intranasal oxytocin and vasopressin on cooperative behavior and associated brain activity in men. Psychoneuroendocrinology 2012; 37: 447–61. 21840129 10.1016/j.psyneuen.2011.07.013 PMC3251702

[25] Meneses A. 5-HT system and cognition. Neurosci Biobehav Rev 1999; 23: 1111–25. 10643820 10.1016/s0149-7634(99)00067-6

[26] Xiao L, Priest MF, Nasenbeny J, Lu T, Kozorovitskiy Y. Biased oxytocinergic modulation of midbrain dopamine systems. Neuron 2017; 95: 368–84.e5. 28669546 10.1016/j.neuron.2017.06.003 PMC7881764

[27] Hung LW, Neuner S, Polepalli JS, Beier KT, Wright M, Walsh JJ, Gating of social reward by oxytocin in the ventral tegmental area. Science 2017; 357: 1406–11. 28963257 10.1126/science.aan4994 PMC6214365

[28] Mottolese R, Redoute J, Costes N, Le Bars D, Sirigu A. Switching brain serotonin with oxytocin. Proc Natl Acad Sci 2014; 111: 8637–42. 24912179 10.1073/pnas.1319810111 PMC4060712

[29] Lefevre A, Richard N, Jazayeri M, Beuriat PA, Fieux S, Zimmer L, Oxytocin and serotonin brain mechanisms in the nonhuman primate. J Neurosci 2017; 37: 6741–50. 28607170 10.1523/JNEUROSCI.0659-17.2017 PMC6596550

[30] Atzil S, Touroutoglou A, Rudy T, Salcedo S, Feldman R, Hooker JM, Dopamine in the medial amygdala network mediates human bonding. Proc Natl Acad Sci U S A 2017; 114: 2361–6. 28193868 10.1073/pnas.1612233114 PMC5338494

[31] Demjaha A, Murray RM, McGuire PK, Kapur S, Howes OD. Dopamine synthesis capacity in patients with treatment-resistant schizophrenia. Am J Psychiatry 2012; 169: 1203–10. 23034655 10.1176/appi.ajp.2012.12010144

[32] Jauhar S, Veronese M, Nour MM, Rogdaki M, Hathway P, Turkheimer FE, Determinants of treatment response in first-episode psychosis: an ^18^F-DOPA PET study. Mol Psychiatry 2019; 24: 1502–12. 29679071 10.1038/s41380-018-0042-4 PMC6331038

[33] Tauscher J, Hussain T, Agid O, Verhoeff NPLG, Wilson AA, Houle S, Equivalent occupancy of dopamine D_1_ and D_2_ receptors with clozapine: differentiation from other atypical antipsychotics. Am J Psychiatry 2004; 161: 1620–5. 15337652 10.1176/appi.ajp.161.9.1620

[34] Mouchlianitis E, McCutcheon R, Howes OD. Brain-imaging studies of treatment-resistant schizophrenia: a systematic review. Lancet Psychiatry 2016; 3: 451–63. 26948188 10.1016/S2215-0366(15)00540-4 PMC5796640

[35] Rilling JK, Demarco AC, Hackett PD, Chen X, Gautam P, Stair S, Sex differences in the neural and behavioral response to intranasal oxytocin and vasopressin during human social interaction. Psychoneuroendocrinology 2014; 39: 237–48. 24157401 10.1016/j.psyneuen.2013.09.022 PMC3842401

[36] de Dreu CKW, Greer LL, van Kleef GA, Shalvi S, Handgraaf MJJ. Oxytocin promotes human ethnocentrism. Proc Natl Acad Sci 2011; 108: 1262–6. 21220339 10.1073/pnas.1015316108 PMC3029708

[37] Zik JB, Roberts DL. The many faces of oxytocin: implications for psychiatry. Psychiatry Res 2015; 226: 31–7. 25619431 10.1016/j.psychres.2014.11.048

